# Biological Significance of the Komodo Dragon’s Tail (*Varanus komodoensis*, Varanidae)

**DOI:** 10.3390/ani14152142

**Published:** 2024-07-23

**Authors:** Anna Tomańska, Martyna Stawinoga, Kacper Szturo, Marzena Styczyńska, Joanna Klećkowska-Nawrot, Maciej Janeczek, Karolina Goździewska-Harłajczuk, Oleksii Melnyk, Tomasz Gębarowski

**Affiliations:** 1 Department of Biostructure and Animal Physiology, Faculty of Veterinary Medicine, Wrocław University of Environmental and Life Sciences, Kożuchowska St. 1, 51-631 Wrocław, Poland; 2 Veterinary Biotechnology Student Science Club “Refectio”, Department of Biostructure and Animal Physiology, Faculty of Veterinary Medicine, Wrocław University of Environmental and Life Sciences, Kożuchowska St. 1, 51-631 Wrocław, Poland; 3 Department of Human Nutrition, Faculty of Biotechnology and Food Science, Wroclaw University of Environmental and Life Sciences, Chełmońskiego St. 37/41, 51-630 Wroclaw, Poland; 4 Department of Animal Anatomy, Histology and Pathomorphology, Faculty of Veterinary Medicine, National University of Life and Environmental Sciences of Ukraine, 03041 Kyiv, Ukraine

**Keywords:** reptile anatomy, monitor lizard, *Varanus komodoensis*

## Abstract

**Simple Summary:**

This study presents morphological research on the tail, with particular emphasis on its skeleton, musculature, and accumulated fat tissue. Additionally, it provides an examination of the spinal cord and anal glands of the Komodo dragon. This project sheds new light on the biological importance of the tail in this lizard, offering insights into conservation implications, which are of utmost importance due to the threat of extinction of this species. As a result of the conducted research is a multifaceted analysis of the tail, with particular emphasis on its numerous biological functions.

**Abstract:**

The Komodo dragon is a unique reptile with an elongated tail that exhibits hitherto unknown adaptations and functions. This tail, composed of 60–86 vertebrae, serves diverse ecological and physiological roles. In juveniles, it is essential for an arboreal lifestyle and balance, while in adults, it functions as a tool for defense and offensive actions. It possesses characteristic haemal arches and a dorsal keel, along with well-developed muscles which enable precise tail control, influencing the Komodo dragon’s maneuverability and directional changes. The tail stores adipose tissue, providing Komodo dragons with the ability to regulate body temperature and independence from other seasonal variations. The tail adipose tissue impacts numerous biochemical processes and may play a crucial role in the animals’ metabolic strategies and reproductive capabilities. Its functions include providing essential mineral compounds for the organism, such as calcium, phosphorus, magnesium, iron, and zinc. Analysing the biochemical composition of tail fat is crucial for understanding the health of Komodo dragons.

## 1. Introduction

The transfer of knowledge from zoo research to field scientists plays a crucial role in the conservation of biodiversity and the management of species maintained ex situ [1] and in situ [2], many of which have recently been granted special status. Understanding their biology is considered a priority, given the ongoing serious biodiversity crisis and the significance of human influence on the new trajectory of evolution. Considering the ongoing or potential “Sixth Mass Extinction” and the fact that the estimated extinction rate disproportionately affects island species [3], the specimen of a deceased female Komodo dragon from the Wrocław Zoo underwent extensive research. Tails serve important functions in animals and exhibit various shapes, with significant morphological diversity [4], and understanding this diversity allows us to comprehend species evolution. The structure of the Komodo dragon’s tail has been meticulously described, and we have indicated possible biological adaptations, considering the existing literature data. We have addressed conservation implications as our findings can directly contribute to its protection.

In this study, we present the micro- and macro-morphological structure of the tail. We began with the dissection of the skeletal system and description of the muscles, including the spinal cord of the caudal vertebrae and cloacal glands, and then conducted detailed examinations of the fat tissue stored in the tail. The primary goal of the research was to understand and describe the general morphology of the tail. After initially describing the skeletal and muscular systems, we conducted a more detailed analysis of the *M. Ilio-ischio-caudalis* and *M. caudofemoralis longus* muscles, focusing on their histological features and morphometry. This examining included evaluating cross-sections and fibre length variations along the length of the tail. During the anatomical studies, the presence of accumulated adipose tissue was observed, and the cloacal glands were dissected. These structures added additional biological significance to the context of the morphological studies, prompting a discussion on the biological importance of the tail. The presented hypothesis regarding the ecological importance of the *Varanus komodoensis* tail required a comprehensive research methodology. This allowed us to establish the connection between anatomy and the tail’s primary functions, further highlighting the role of the tail in fat storage. This fat tissue can be used to assess various environmental parameters and the animal’s well-being. The project was realised through diagnostic imaging, histological, biochemical, and toxicological examinations; analysis of data collected from veterinarians and caregivers at selected zoological gardens; video and real-time material observations; and a literature review, along with comparative material analysis.

## 2. Materials and Methods

### 2.1. Animals

This study was conducted on a single specimen of a 7-year-old female Komodo dragon (*Varanus komodoensis* [Ouwens 1912]) obtained from the Wrocław Zoological Garden (Wrocław, Poland), acquired after natural death. The specimen was preserved by complete immersion in a formalin tank. The female was weighed and measured, with a mass of 30 kg, a snout-to-vent length (SVL) of 93 cm, a head length (HL) of 18 cm, and an ear opening–snout distance (EOS) of 15.4 cm. The tail length measured 107.8 cm (TL). The circumference of the tail at the cloacal region was 32.3 cm; at the mid-section, it was 20.1 cm; and at the tip, it had a circumference of 3.0 cm. Zoometric measurements were taken using a flexible tape measure, with two independent measurements performed and confirmed using dimensions collected with a standardised archaeological ruler (made of synthetic paper). The results were averaged and rounded to one decimal place. The fat accumulated in the tail had a total mass of 487 g. The research was conducted by the Department of Biostructure and Animal Physiology, Faculty of Veterinary Medicine, in collaboration with the Department of Human Nutrition, Faculty of Biotechnology and Food Science. Both departments are part of the Wrocław University of Environmental and Life Sciences (Wrocław, Poland).

For comparative studies, the skeleton of a Komodo dragon from the collections of the Archaeology Laboratory and the Nature Museum of Wrocław University of Environmental and Life Sciences (Wrocław, Poland; no catalog number assigned) was used. The skeletal data also originated from an individual from Senckenberg Natural History Museum (Frankfurt am Main, Germany; specimen no SMF 57555), and five specimens from the Skeletons: Museum of Osteology (Oklahoma City, United States, catalog no: 3524, 4320, 6690, 8096, 8098). The research study on the lizard’s tail is part of extensive ongoing research on this species. Following all stages of the tail analysis, tissue remnants were initially removed, and the bones were boiled with detergent. Subsequently, the bones were macerated in a perhydrol solution and dried. The tail skeleton was then arranged and mounted on a frame. The material is now part of the university’s anatomical collection and will be catalogued and preserved in its entirety.

To broaden the scope of the research, we included two living Komodo dragons from the Poznań Zoological Garden (Poznań, Poland), and two other *Varanidae* species (*Varanus niloticus* and *V. prasinus*) from the Kiev Zoological Garden (Kiev, Ukraine). Information was gathered from zookeepers via surveys, focusing on their direct experiences and routine veterinary examination results. Additionally, the study involved real-time observation and an analysis of publicly available online video footage.

### 2.2. Experimental Methods and Procedures

The position of the tail was assessed, and its metric data in relation to the animal’s body were analysed, with a detailed description of its characteristics. X-ray images were captured and interpreted using a CR 7 VET scanner (iM3, Lane Cove, NSW, Australia) and Vet-Examplus 9.3.0 software (iM3 Pty Ltd., Lane Cove, NSW, Australia). The tail, along with the entire animal, was weighed, and the individual’s documentation was reviewed. All caudal vertebrae were counted on the radiographs of the examined specimen and the skeleton of an individual in the collection of the Archaeology Laboratory and the Nature Museum of Wrocław University of Environmental and Life Sciences (Wrocław, Poland). Requests for data on the tail skeleton were also sent to the Senckenberg Natural History Museum (Frankfurt, Germany) and the Skeletons: Museum of Osteology in Oklahoma City (United States). Subsequently, the tail was skinned, and the features of the scales and subcutaneous tissue were assessed. The tail was then sectioned to obtain cross-sectional slices. During these procedures, the variability of anatomical structures was examined, with particular attention to skeletal elements and muscles, and fat storage was also observed. The musculature of the tail was described in detail, and its variation concerning its location in the cross-sections of individual segments was examined.

Samples of the *M. Ilio-ischio-caudalis* and *M. caudofemoralis longus* muscles were extracted. These muscles were fixed in formalin, processed through a series of alcohols and aldehydes, embedded in paraffin blocks, and then sliced into 5–10 µm sections, resulting in three basic slide preparations. Hematoxylin and eosin (H&E) staining was performed and slides were evaluated under a microscope. Cross-sectional fibre measurements and fibre length were assessed for each of the preparations (N = 100 measurements for each of the individual locations, with standard deviation indicated as ±SD). Measurements of the specified muscles were conducted for the initial segment (where the tail circumference measured approximately 30 cm), the middle segment (approximately 20 cm in circumference), and the end segment (approximately 10 cm in circumference). Mean lengths and widths of the cross-sectional muscle fibres were calculated, along with their respective standard deviations (±SDs).

During the sectioning of the tail, all the accumulated fat deposits were located, selected, weighed, and sent for in-depth analysis to the Food Research Laboratory in the Department of Human Nutrition at Wrocław University of Environmental and Life Sciences (Wrocław, Poland). All fat was extracted along the length of the tail, and four representative samples were taken from the collective container. The purpose of this analysis was to determine the fatty acid profile and measure heavy metal levels. The fatty acid profile determination was conducted using a mixture of 14% BF3, 0.4 M NaOH (methanolic solution), NaCl (saturated solution), and hexane. The sample was heated and cooled before extraction and analysis on a GC Agilent 7820A with a Zebron ZB-WAX column. The carrier gas used was helium, and the Flame Ionisation Detector (FID) settings were specified. Heavy metal measurement was carried out using mineralisation of the samples was carried out using a “wet” process within a closed microwave system, which involved adding 5 cm^3^ of concentrated nitric acid V (CTC—reagent grade) and 1 cm^3^ of concentrated hydrogen peroxide to a portion of the sample. Sample quantities ranged from 0.1 g to 0.5 g. Subsequently, the samples underwent mineralisation in the MARS 5 microwave sample preparation system. The resulting mineralisations were quantitatively transferred to 10 cm^3^ measuring vessels, after which they were diluted with redistilled water. The determination of calcium, sodium, potassium, lead, cadmium, zinc, iron, magnesium, and copper content was performed using atomic emission spectrometry with an acetylene/air flame. Phosphorus content was determined using a spectrophotometric method. The mineralisation processes adhered to relevant standards. The measurement uncertainty was estimated at 5%.

Histological evaluation of the fat tissue sample was conducted using the same method as for the muscles, involving sectioning the paraffin block slices to a thickness of 5 µm and measuring adipocyte diameter in three slide preparations (N = 100 measurements for each). The mean and standard deviation (±SD) were calculated for all results.

To better understand the possible biological function of adipose tissue, data from blood tests available in the literature and from selected zoos were taken into account. This information was analysed from various varanid and other species (*Varanus komodoensis*, *Varanus niloticus*, *Varanus prasinus*, *Varanus exanthematicus*, *Crocodylus intermedius*, *Iguana i. igauana*). Based on a review of the literature and data from publications, lipid profiles, red and white blood cell parameters, and liver enzymes were collected. Additionally, the lipid profile composition of adipose tissue in the tail of a Nile crocodile (*Crocodylus niloticus*) and in the hump and tail of a Bactrian camel (*Camelus bactrianus*) was also compared to that of the Komodo dragon.

After removing the fat tissue from the tail, the bony skeletal elements in the detached tail were described in detail. Next, at the level of the 6th tail vertebra, the spinal cord was extracted, fixed in formalin, processed through alcohol and aldehyde, embedded in paraffin, sliced into sections ranging from 4 to 8 µm in thickness, and stained with H&E for further microscopic evaluation.

The study also encompassed the paired cloacal glands. Their anatomical structure was described, and they were entirely excised, fixed in formalin, processed in alcohol and aldehyde, embedded in paraffin, sectioned to a thickness of 5–10 µm, and stained with H&E. The resulting slides were evaluated microscopically.

Photographic documentation was created using a NIKON D3100 camera with an 18–55 VR lens. Microscopic analysis of the slides was conducted using a Leica DM4000B microscope with a DCF345 FX camera (Leica Microsystems, Wetzlar, Germany). Illustrations were prepared using a WACOM One S tablet in Adobe Illustrator (Adobe Inc., 2019, San Jose, CA, USA).

After collecting all research results and analyzing the available literature, two survey studies were conducted with caretakers from the Poznań Zoo (Poznań, Poland) and the Kyiv Zoo (Kyiv, Ukraine). These studies aimed to gather data related to husbandry, conservation, and behavioural issues, complementing the context of the possible biological functions of the tail.

Tail vertebrae were cleared of all tissues, boiled, and left in a perhydrol solution for whitening. Subsequently, they were arranged according to anatomical descriptions and collected documentation, then mounted on a framework to assemble the complete skeleton upon completion of all procedures.

## 3. Results

**Figure 1:**
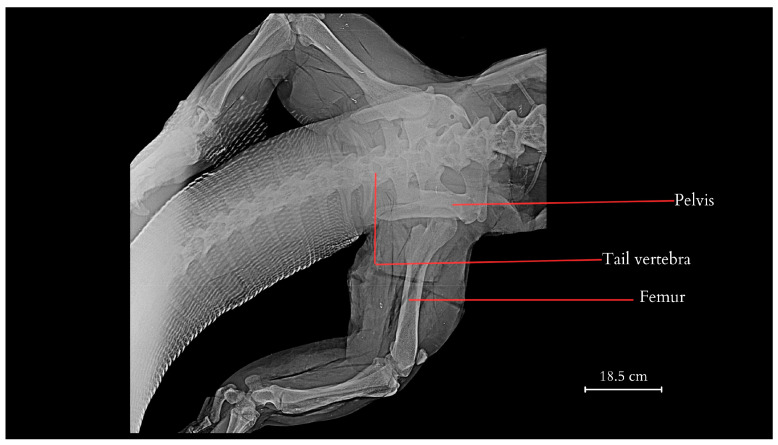
Dorsoventral projection of the pelvis, lumbar vertebrae, partial caudal vertebrae of the tail, and femur bones of the pelvic limbs of the Komodo dragon (X-rays).

**Figure 2:**
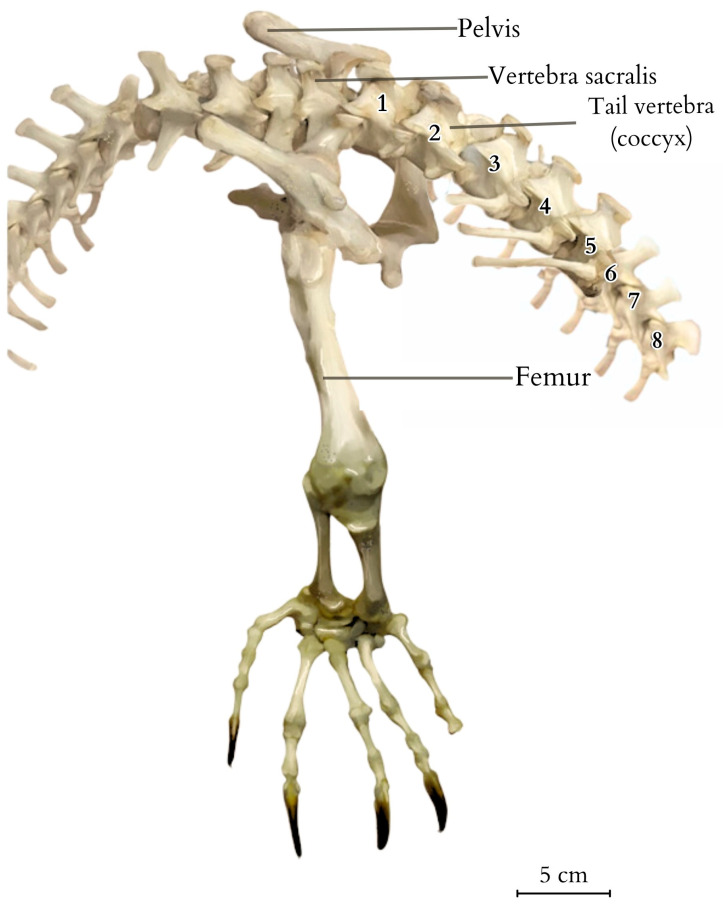
The caudal vertebrae, with the first one lacking haemal arch (vertebrae numbered as 1–8). The visible connection of three different segments of the spine, the suspension of the pelvis, and the attachment of the limbs through the pelvic girdle joints. Illustration based on the anatomical examination and photographic documentation of the studied specimen and a comparative skeleton from the collections of the Archaeology Laboratory and the Nature Museum of Wrocław University of Environmental and Life Sciences (Wrocław, Poland), created by Anna Tomańska.

**Figure 3:**
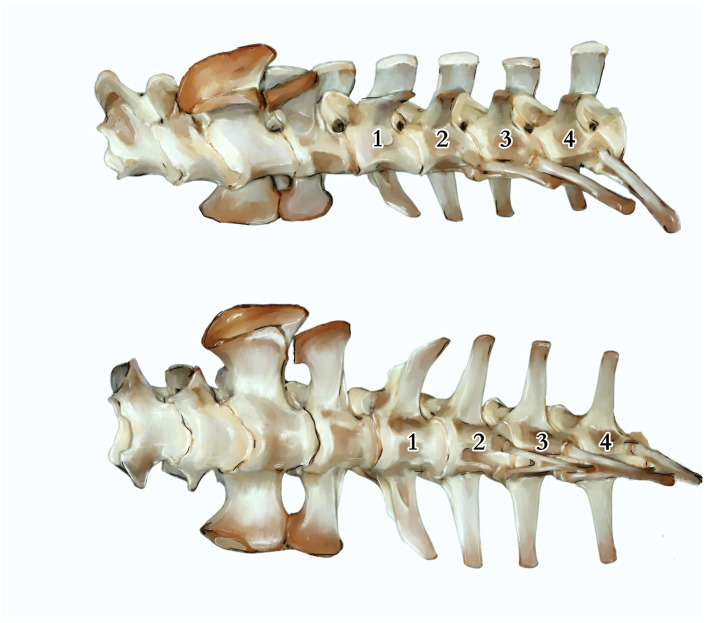
The last lumbar vertebrae, sacral vertebrae, and initial caudal vertebrae (numbered 1–4), along with the visible haemal arches of specimen SMF 57555 from Senckenberg Natural History Museum (Frankfurt, Germany). Based on photographs, courtesy of Gunther Köhler, illustration by Anna Tomańska.

The tail measured 107.8 cm in TL (Tail Length, measured from the tip to the cloacal opening). It was covered with hard and sharp scales constructed of keratinocytes that extended deep into the centres of the epithelial tissue and the lower layers of the dermis. The skin exhibited significant hardness and thickness, with the presence of ossification centres. The thickness gradually decreased towards the end of the tail, being thickest and hardest in the upper part above the spinous processes. Along the tail’s course, it was observed that the spinous processes began to protrude above the muscle surface, connecting directly to the skin through connective tissue. Subcutaneous tissue was a noticeably reduced layer of connective tissue, essentially devoid of adipose tissue. Some of the scales exhibited morphological variations. For example, in the anal region, they took on a hexagonal and heptagonal shape with a rounder, brighter centre. The scales displayed distinct radiographic shading, forming a regular pattern encircling the tail (Figure 1), giving the tail a smooth but non-uniform colouration with visible bands of darker and lighter hues. The tail’s skeleton consisted of a total of 68 vertebrae, which varied in anatomical structure and size. The first tail vertebrae lacked a haemal arch (Figure 2 and Figure 3). The second caudal vertebrae had a haemal arch oriented at an acute angle towards the base of the vertebra.

**Figure 4:**
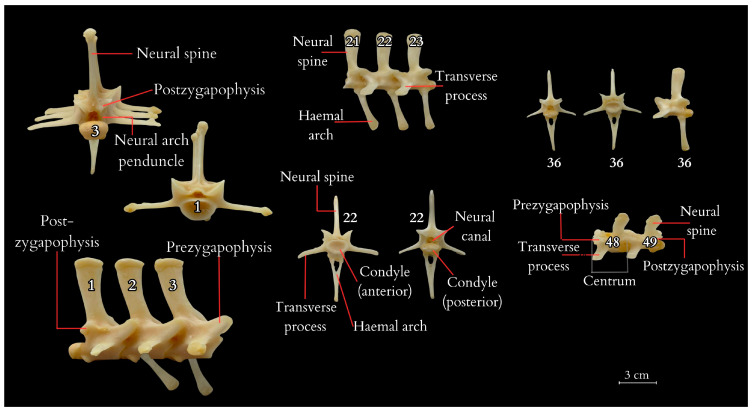
Tail vertebrae of the Komodo dragon obtained from the carcass of the studied specimen after dissection and preservation. Among the anatomical structures described on the vertebrae are neural spine, neural arch peduncle, prezygapophysis, postzygapophysis, transverse process, haemal arch, condyle (anterior and posterior), centrum. 1–3, 21–23, 36, 48, 49—tail vertebrae numbering.

**Figure 5:**
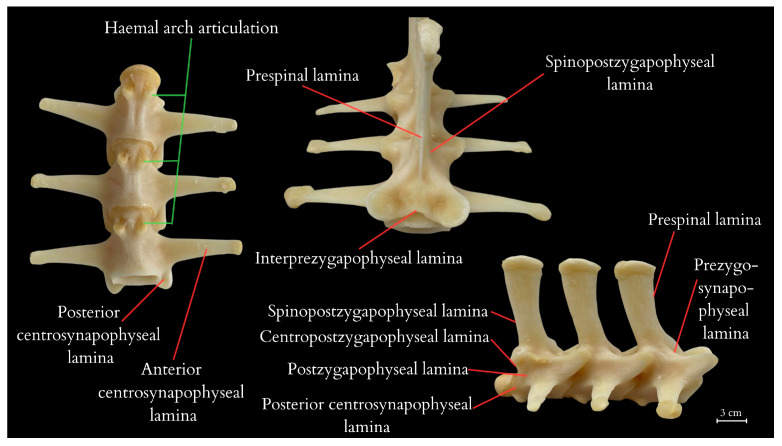
Laminae in caudal vertebrae, haemal arch articulations of the Komodo dragon obtained from the carcass of the studied specimen. The anatomical structures described in this figure include, in sequential order, prespinal lamina, prezygosynapophyseal lamina, centropostzygapophyseal lamina, postzygapophyseal lamina, posterior centrosynapophyseal lamina, spinopostzygapophyseal lamina, and anterior centrosynapophyseal lamina.

**Figure 6:**
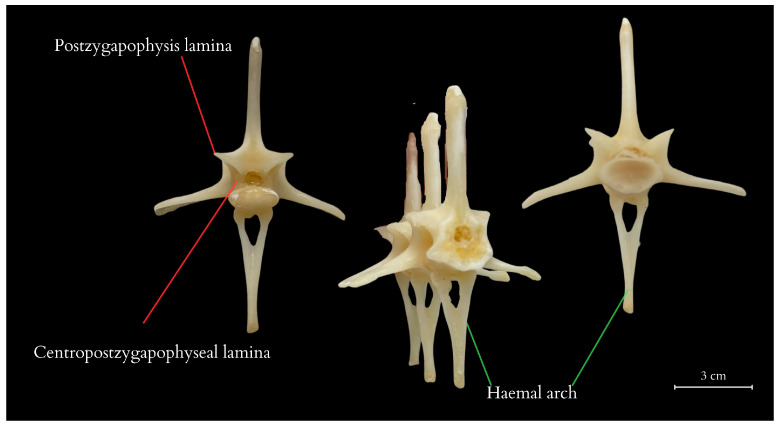
Vertebral laminae in caudal vertebrae and haemal arch of the Komodo dragon obtained from the carcass of the studied specimen. Additionally, postzygapophysis lamina and centropostzygapophyseal lamina were also described.

The tail vertebrae exhibit differentiation into the proximal, transitional, and distal regions of the tail, serving, among other functions, as a skeletal support for the well-developed muscles and accumulated fat. The central element of each vertebra is the vertebral body, which forms the main part of the vertebra. On top of each vertebra is the neural spine, providing a broad attachment site for muscles and ligaments. The transverse processes are visible on the sides of the vertebra, serving as additional attachment points for muscles. At the anterior part of the vertebra is the prezygapophysis and at the posterior part is the postzygapophysis, which facilitate articulation with adjacent vertebrae, essential for proper tail mobility. Inferior to the vertebra is the haemal arch, a structure that protects the blood vessels traversing this region. Within the vertebra is the neural canal, enclosing the spinal cord. (Figure 4 and Figure 5). It was observed that the spinous processes in a juvenile (approximately 3–5 years old) from the archaeological collection were significantly shorter (Figure 2) than in the dissected female (Figure 4). The first two tail vertebrae have reduced transverse processes. Starting from the second tail vertebrae, they develop haemal arches, forming the haemal arch in which a large blood vessel runs. The haemal arch is open and Y-shaped from a cranial view, and it has a curved structure from a lateral view, connecting with the tail vertebra through two articulations (Figure 5 and Figure 6) [5]. The spinal cord is located in the neural canal, with blood vessels not running alongside the nerves but instead being shielded by haemal arches. The tail spine is surrounded by well-developed muscle masses, with the highest contribution in the proximal part. In the transitional part, there is an increasing presence of adipose tissue, while in the distal section, the muscles are reduced and adipose tissue is absent. The distal part of the tail is primarily composed of connective tissue, connecting the vertebrae to the hard scales.

**Figure 7:**
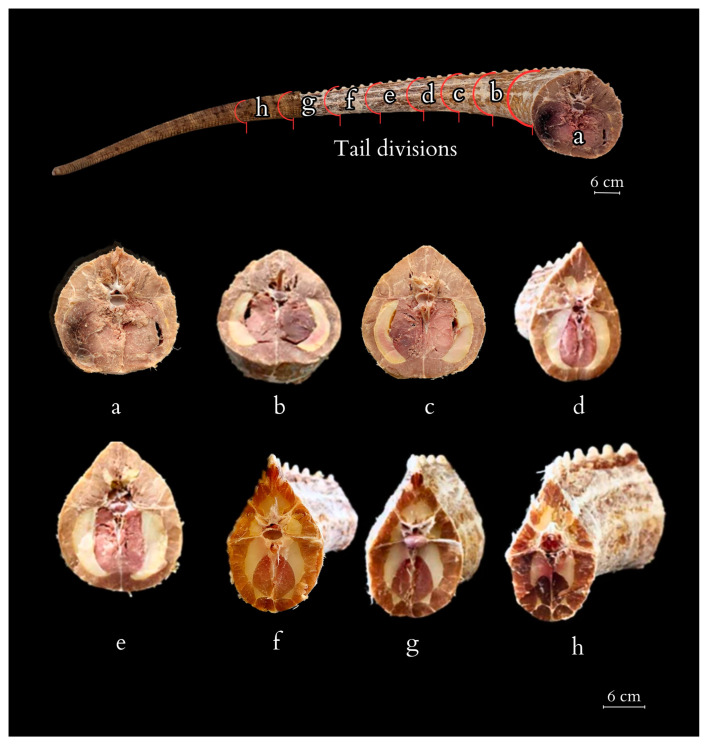
Tail of the Komodo dragon obtained from the carcass of the studied specimen, along with 8 transverse cross-sections highlighting the stored adipose tissue and muscle architecture (a–h indicate the numbering of consecutive transverse sections).

**Figure 8:**
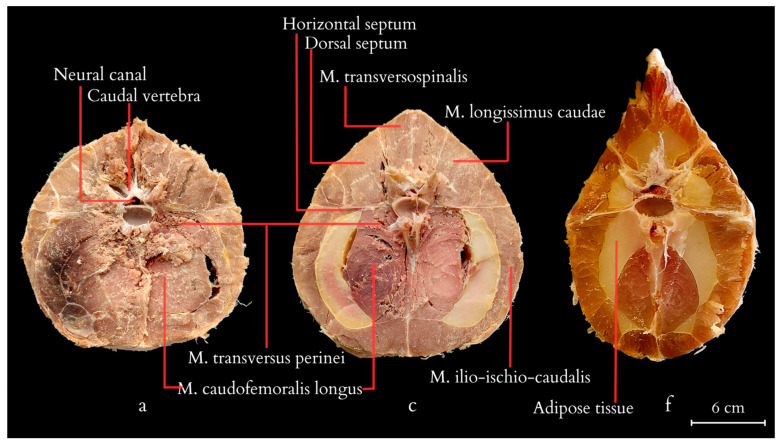
Anatomic structure of the tail muscles (transversal view; a, c, f—cross-section numbering). The anatomical structures visualised include the neural canal, caudal vertebra, horizontal and dorsal septum, adipose tissue, and muscles: the *M. caudofemoralis longus*, the *M. transversus perine*i, the *M. transversospinalis*, the *M. longissimus caudae*, the *M. ilio-ischio-caudalis*.

On transverse cross-sections of the tail, the muscle architecture and the location of accumulated adipose tissue are visible (Figure 7). Fat tissue begins to deposit behind the cloacal region, initially forming thin layers between different muscle sections within the septa that separate the muscle masses. It accumulates in dense layers, without integrating into the architecture of the muscle fibres themselves. Initially, fat layers accumulate between the *M. caudofemoralis longus* and *M. Ilio-ischio-caudalis*, forming bands of adipose tissue on the sides of the tail resembling crescents located under the transverse processes of the vertebrae. Further down the tail, adipose tissue begins to accumulate closer to the spinous processes and above the transverse processes. In cross-section, the muscles reveal a radial arrangement relative to the vertebrae and fat tissue. As the disproportion between fat and muscles increases, a change in the architecture of muscle fibres becomes noticeable, with a regular radial arrangement of bands demarcated by tendons (Figure 8). The muscle structure gradually diminishes across the entire cross-section, with the scales closely adhering to the vertebrae towards the end, causing the projecting arch and body processes to lie beneath the body of a single flat scale. Loose deposits of adipose tissue are found in the closer-to-pelvis region, forming irregular bundles subcutaneously. Apart from that, fat was not observed in a disorderly manner and outside of the accumulated layer.

**Figure 9:**
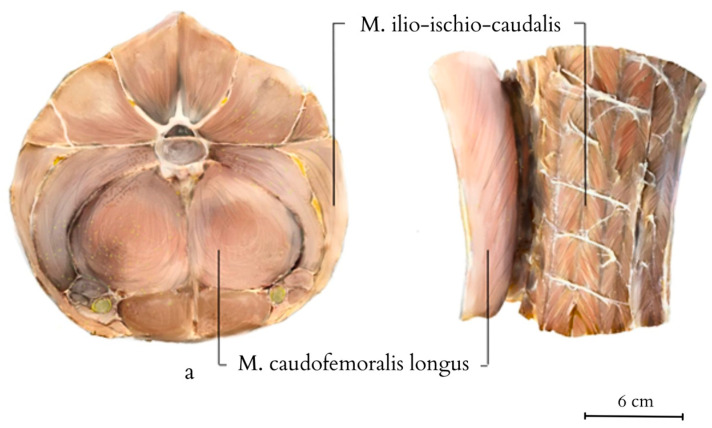
Cross-section a, transversal view, and in the sagittal plane of the *M. Ilio-ischio-caudalis*. Also described is the *M. caudofemoralis longus* muscle. Illustration by Anna Tomańska.

In the proximal part of the tail, all the muscles are most strongly developed. The largest muscle in cross-section is the *M. caudofemoralis longus*, along with the *M. Ilio-ischio-caudalis*. The *M. caudofemoralis longus* forms longitudinally running muscle fibres. The *M. Ilio-ischio-caudalis*, on the other hand, creates alternating bands of oblique muscle fibres separated by connective tissue. They form a pattern resembling a tuning fork when viewed relative to each other (Figure 8 and Figure 9). Nerve fibres regularly traverse across the main muscle bands in a transverse direction.

Additionally, vertebral laminae were identified, some of which are present only in species of large sizes, which might be of taxonomic value in future studies of ontogenetic variability (Figure 5) [6].

**Figure 10:**
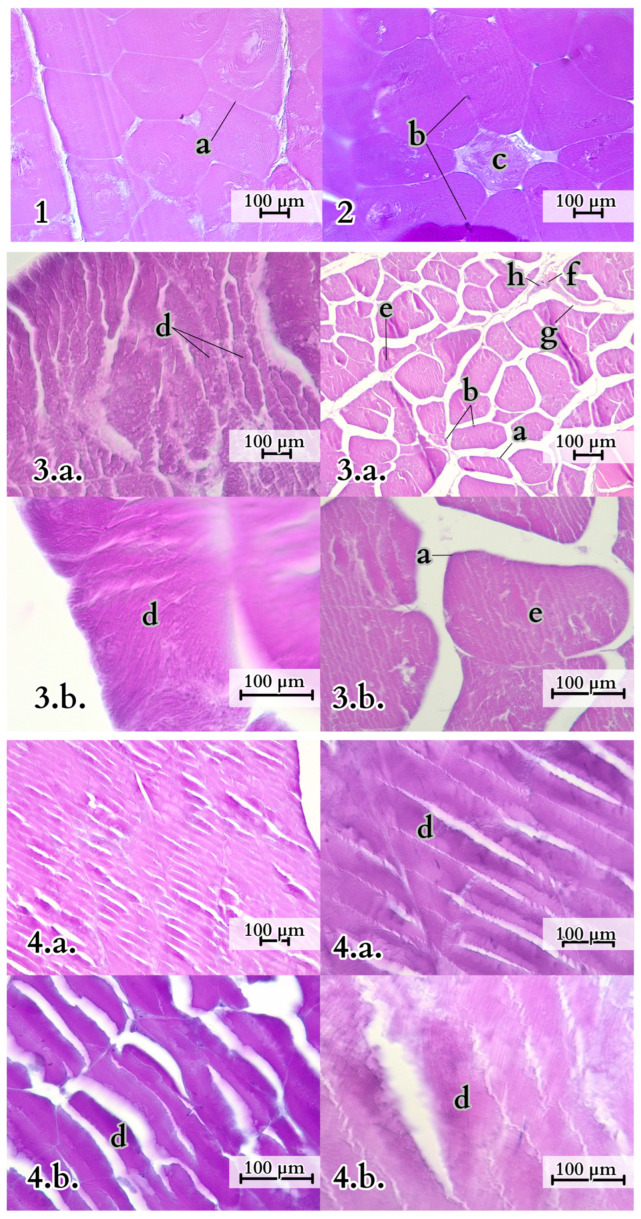
Variation in muscle fibres of selected muscles in the Komodo dragon’s tail. 1—*M. ilio-ischio-caudalis* in the proximal part of the tail (cross-section), 2—*M. caudofemoralis longus* in the proximal part of the tail (cross-section), 3—*M. caudofemoralis longus* in both transverse (cross-section 3.a.) and distal parts (cross section 3.b.), 4—*M. Ilio-ischio-caudalis* in tranverse and distal part of the tail (cross-section, 4.a.) and transverse and distal part (longitudinal section, 4.b.). Key structures identified within the muscle tissues include a—sarcolemma (muscle cell membrane), b—myocyte nucleus, c—nerve, d—muscle fibre, e—myocyte (muscle cell), f—fibroblast, g—perimysium, h—capillaries. H&E examination with microscopic analysis was conducted using a Leica DM400B microscope with a Leica DCF345 FX camera.

The tail muscles display visible striations and have distinct architectures. In the proximal part of the tail, there is close adjacency of muscle fibres of the *M. caudofemoralis longus* and *M. Ilio-ischio-caudalis*. In the cross-section of the *M. Ilio-ischio-caudalis*, the fibres are regular (usually hexagonal, fitting into an oval shape) and gathered in bundles, with perimysium separating them into compartments running parallel to each other. The *M. caudofemoralis longus* has pentagonal, more elongated fibres. Their diameter, measured from one to the other, is usually slightly longer. In the transverse and distal parts of the tail, the bundles of muscle fibres of this muscle are more spaced apart, and individual fibres lose their spiral arrangement in favour of a parallel orientation, displaying irregular branching. On the other hand, in the case of *M. Ilio-ischio-caudalis*, in the transverse and distal sections, the fibre arrangement is reorganised. They are arranged in bundles running parallel to each other, and the fibres themselves lie obliquely to their course. The fibres are visibly shorter and, both in the cross-section and longitudinal view, fit into a rectangle. Adipocytes were not observed in the histological images of the muscle specimens (Figure 10).

**Figure 11:**
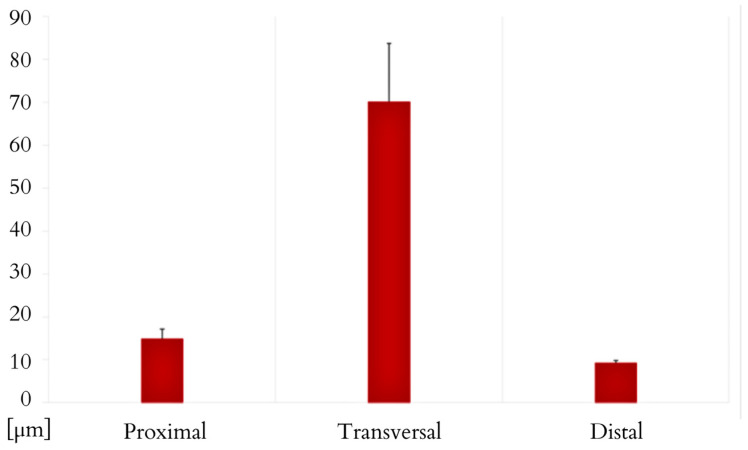
Morphometric data for the width of muscle fibres in the cross-section of the *M. ilio-ischio-caudalis*. Measured in the proximal, transverse, and distal parts of the tail (average obtained from 3 samples, with N = 100 measurements for each, including minimum, maximum, and mean values with standard deviation ± SD).

**Figure 12:**
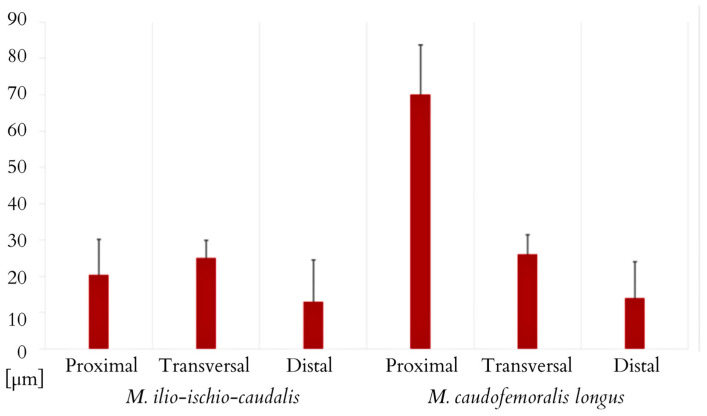
Morphometric data for the length of muscle fibres for both M. ilio-ischio-caudalis and M. caudofemoralis longus (average of N = 100 measurements for each of the 3 samples, including minimum, maximum, mean values, and standard deviation ± SD).

**Figure 13:**
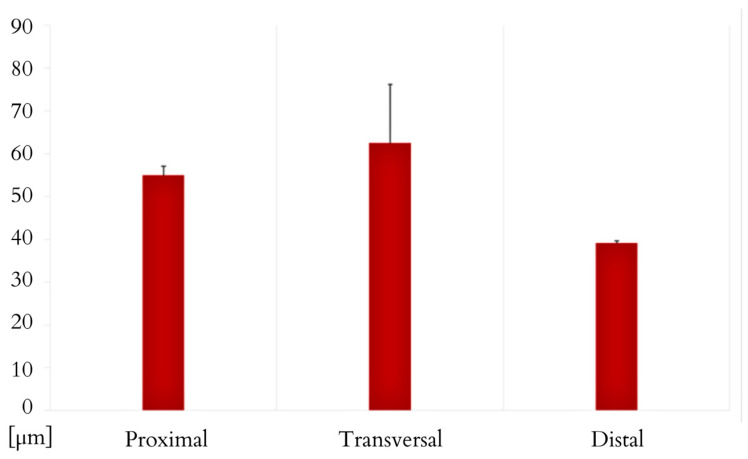
Morphometric data for the width of muscle fibres in the cross-section of the M. caudofemoralis. Measured in the proximal, transverse, and distal parts of the tail (average obtained from 3 samples, with N = 100 measurements for each, including minimum, maximum, mean values, and standard deviation ± SD).

The *M. ilio-ischio-caudalis* fibres exhibit their greatest cross-sectional width in the middle part of the tail, while in the proximal and distal parts, they are similar in width. Their width along the entire length of the tail varies from 6.17 to 130.76 µm. The standard deviation of the collected data indicates that the fibres are most diverse in terms of width in the middle part. This muscle also shows a similar trend in terms of the length of muscle fibres; they are longest in the transverse part but most diverse in the distal part (Figure 11 and Figure 12). In the proximal part, the *M. caudofemoralis longus* has long fibres with the most varied cross-sectional width; in the transverse part, they are the widest, ranging from 8.25 to 113.39 µm along the entire tail (Figure 12 and Figure 13).

**Figure 14:**
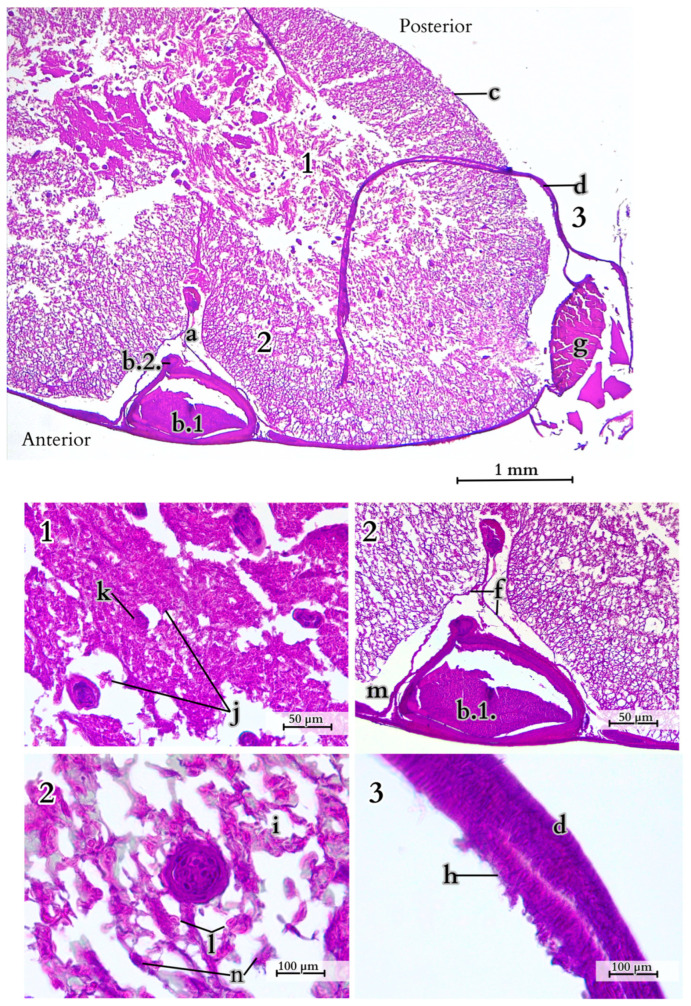
Spinal cord organisation (transverse section). 1—grey matter, 2—white matter, 3—meninges, a—anterior white commissure, b.1—anterior spinal vein, b.2—anterior spinal artery, c—pia mater, d—dura mater, f—linea splendens, g—ventral nerve root, h—mesothelium, i—nerve fibre tissue, j—motor neurons, k—Nissl bodies, l—astrocytes, m—subdural space, n—neurons. H&E examination with microscopic analysis was conducted using a Leica DM400B microscope with a Leica DCF345 FX camera.

The spinal cord in the tail section exhibits a greater proportion of grey matter surrounded by the pia mater and the dura mater. White matter is predominant, with well-developed anterior grey horns. In the anterior region, there is a large vein and smaller arteries or ganglia. In the lateral parts, there are prominent ventral nerve roots (Figure 14).

**Figure 15:**
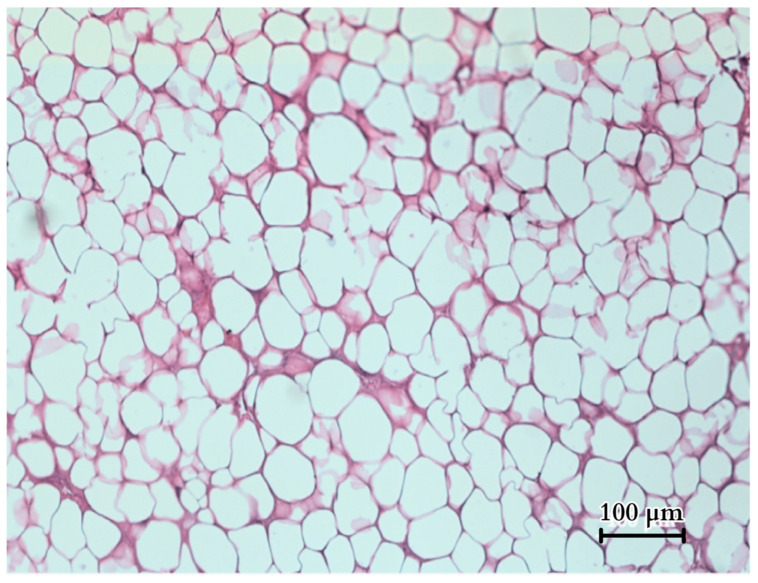
Histological image of the tail fatty tissue (H&E).

Histology of the fatty tissue reveals adipocytes (Figure 15), with a mean diameter of 54.37 µm (ranging from 35.50 to 85.51 µm cell diameter, with a standard deviation of 11.08 ± SD, based on a sample size of N = 100). The fatty tissue exhibited a light yellow colour and a compact, firm consistency and formed solid layers separated by connective tissue within the septa that separated the tail muscles. In total, the fat had a mass of 487 g. In this species, fat tissue is minimally present in other regions of the body, as evidenced by the small abdominal fat deposits seen during dissection. As a result, the tail serves as the primary location for the majority of the body’s adipose reserves.

For the analysed 1 µL sample of the collected fat, the overall fat content was 88.64%. The fat is composed of saturated fatty acids (44.34%), including monounsaturated (45.09%) and polyunsaturated (11.33%) fatty acids. The dominant are palmitic, oleic, linoleic, and stearic fatty acids. The fat also contains various minerals, including sodium, potassium, phosphorus, calcium, copper, magnesium, iron, zinc, cadmium, mercury, lead, and zinc, as determined by the biochemical and toxicological analysis (Table 1 and Table 2).

The cloacal glands, with openings approximately 2.5 cm wide, are symmetrically located on both sides above the cloacal opening, surrounded by three smaller muscles. They are paired glands, one on each side of the cloaca. The outlet ducts, emerging from the glandular part, are situated beyond the region of the external anal sphincter muscle, within its cutaneous area, but they lead externally beyond the rectal opening. A small layer of fatty tissue is deposited on the rear wall of the gland.

**Figure 16:**
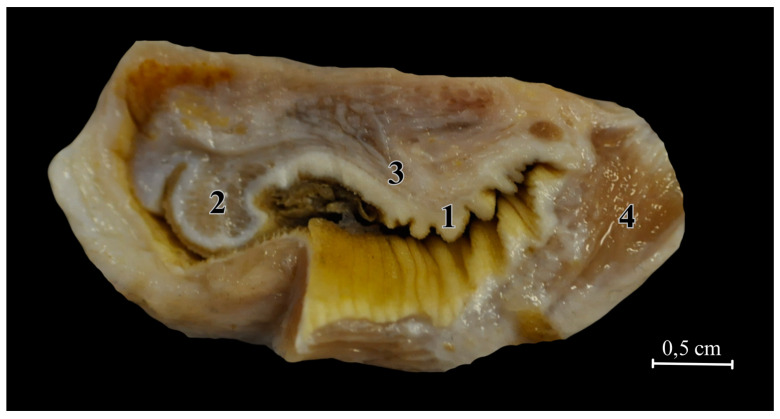
Cloacal gland in the anal region. 1—the outlets of the glandular ducts, 2—the proper gland, 3—outlet ducts, 4—muscles.

In the collecting part of the outlet ducts, there is a constant, brittle holocrine secretion with a dark honey-like colour (Figure 16). The glandular mass is concentrated in the compound areas of the gland. Three primary lobes empty into common outlet ducts, and they are surrounded by connective and muscular tissue, including a well-developed muscular layer (tunica muscularis), which terminates in a stratified squamous epithelium with a cornified surface, covered with a layer of mucus and oily secretion.

The outlet ducts extend in the form of bands composed of columnar cells with well-stained cell nuclei, and some of them have longer invaginations. The duct openings are arranged with folds and small protrusions that conform to the shape of the skin part of the glands, which is significantly folded, increasing the secretory surface area.

This skin section encloses the entire gland in the shape of the letter “C” and forms a sinus that collects the produced secretion. In the region bordering the main compound glandular part, skin formations resembling small needles develop, all of which are located on the cross-striated skeletal muscles.

**Figure 17:**
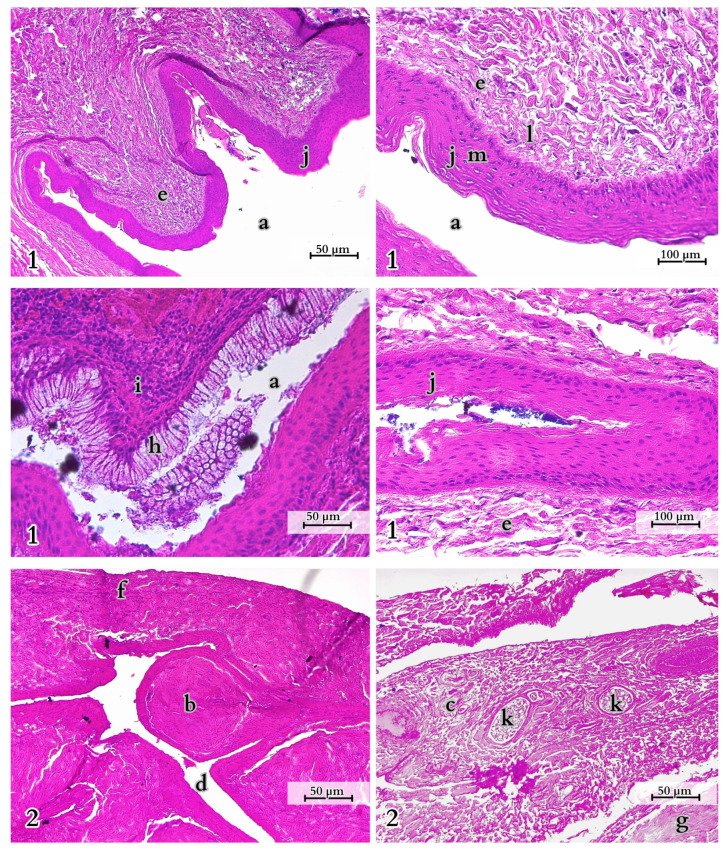
Morphology of rectal glands (cross-section). 1—the outlets of the glandular ducts, 2—the proper gland. a—anal orifice, b—holocrine gland, c—apocrine gland, d—trabeculum, e—connective tissue, f—connective tissue capsule, g—muscle, h—absorptive cells, i—epithelium, j—nonkeratinised stratified squamous epithelium, k—vein, l—lamina propria, m—epithelium. H&E staining with microscopic analysis conducted using a Leica DM4000B with a Leica DCF345 FX camera.

Within the glandular epithelium, variations in the basilar surface and simple columnar epithelium (glandular cells) can be distinguished. In the histological section, numerous interlobular ducts, interlobular connective tissue septa, and outlet ducts can be identified. These glands contain both serous and mucous secretory units (Figure 17).

## 4. Discussion

### 4.1. Overview of the Morphology and Features of the Tail

The tail of the Komodo dragon is relatively long, constituting 54% of the total length of the animal. A similar observation was made by Kalman [7]. In herpetological museum collections, these values reached 46%, 46%, and 50% (Senckenberg Natural History Museum, Frankfurt am Main), approximately 46% of SLT (snout to tail length) at the British Museum in London [8]. In juveniles, TL (tail length) is longer than SLV (snout to vent length), and as the animals grow, the length ratio evens out [9]. These data do not affect the determination of phenotypic sex, as external morphology encompasses only slight differences in size and colouration [10]. However, no such typical external differences have been identified within the tail. So far, only a relationship between the arrangement of pre-cloacal scales and gender has been recognised, along with the presence of hemiclitoreal sacs in females where males have hemipenes [11]. While this study does not address the detailed morphology of the skin, we acknowledge its importance. This aspect is being examined in a separate research project, which will provide further insights in due course. Characteristic oval and six- and seven-sided scales with a flat centre are found on the lower part of the tail and are worth investigating for the possible presence of femoral gland openings. The significance of such structures in chemical signalling has already been noted in many lizards [12]. External morphology features hard and sharp scales, creating a uniform smooth surface with alternating brown and beige stripes irregularly interspersed with dark brown and black spots. This colouration is most pronounced in the tail and is a species-specific trait, although no research has yet been conducted on variations in colouration based on age, gender, or subpopulations. The hardness and durability of the outer layer stem from the deep osteodermin in the dermal layer and mineralised collagen [12]. This is of great importance because the skin in the dorsal part of the tail, where muscle tissue is greatly reduced, tightly connects to the bones on connective tissue basis. In these areas, it is also much less movable than in other parts of the body. The tail, covered with rigid scales, posed considerable difficulty for sectioning; this only became feasible after the skin was removed. The scales contributed to the tail’s sharpness, indicating a protective adaptation to shield the underlying tissues and enhance its ability to inflict injuries during combat.

The cross-section of the tail changes from initially oval (at the base) through tear-shaped to triangular (at its tip). The oval cross-section at the base of the Komodo dragon’s tail indicates a greater contribution to body balance, and, only along the tail’s length, there is a noticeable change to an oval shape with a dorsal hump, which predisposes the tail to perform side-to-side movements.

### 4.2. Anatomical Analysis of the Caudal Vertebrae, Species-Specific Characteristics

In lizards, the tail vertebrae are linearly differentiated, and changes within the tail may be significant and are typical in terms of taxonomic lineages but to a lesser extent relate to tail function, as Etheridge suggests [13]. In *V. komodoensis*, this differentiation is visible in terms of the number, shape, and orientation of processes. It has 60–86 total tail vertebrae (*ossa vertebrae caudales*). In varanids, noticeable variability in postsacral vertebrae is found—*V. salvator* has 54 such bones [14], *V. acanthurus* 81–95, *V. brevicauda* 47–66.5, *V. eremius* 110, *V. gouldii* 109–115, *V. indicus* 119–128, *V. mertensi* 114–118, *V. primordius* 74–81, *V. storni* 73–78, *V. timorensis* 102–107, and *V. Darius* 137–143 [15]. The number of tail vertebrae in the Komodo dragon may correspond to the need to provide balance for its heavy trunk, neck, and head, as well as a place for tail muscle attachment, and this could be an implication for the giant size of the animal and the specificity of its growth and gait. Although this does not agree with Collar et al., who showed that tail length has the lowest correlation with body size among *Varanus* species, and a relatively long tail is correlated with arboreal and rock-dwelling species [16].

The first tail vertebrae have strong and wide bodies, their transverse processes are more horizontal, and the neural arch is wider than in the other vertebrae. In the transversal part of the tail, the bodies decrease in width, and the transverse processes are positioned vertically relative to the body or slightly inclined dorsally. Distal tail vertebrae have significantly elongated, massive vertebral bodies and even more inclined neural arches. Some of the tail vertebrae of *V. komodoensis* exhibited neurocentral suture closure. Tail vertebrae, except the first one, are equipped with haemal arches, which are not present in all animal species, even those with tails. This observation was made during the dissection of a female Komodo dragon, as well as from a specimen housed in the collections of the Archeology Laboratory and the Nature Museum of Wrocław University of Environmental and Life Sciences (Wrocław, Poland), and in the specimen SMF 57555 from the Senckenberg Natural History Museum (Germany) collection, and was also confirmed in the collection of five skeletons (No. 3524, 4320, 6690, 8096, and 8098) at the Skeletons: Museum of Osteology in Oklahoma City (United States).

Haemal arch observation is important in investigating phylogenetic relationships of dinosaurs [5]. In various animals such as dinosaurs, snakes, reptiles (e.g., crocodiles) [17,18], and fish [19], this bone is responsible for maintaining tail stability, balance, and precise tail movements. This bone is present in various lizard species [20,21], such as iguanas [20], anoles [22], or agamids [22,23], allowing these lizards to adapt to their ecological niches.

The lack of the haemal arch on the first vertebrae allows for greater tail movement to the left and right, its elevation, and proximity to the body, significantly facilitating egg laying, defecation, copulation, and combat. By increasing the anal opening space, it reduces the likelihood of vessel and nerve damage and allows for the elimination of large fecal masses and eggs. During excrement, the lizard raises its tail. As with *V. marathonensis*, the first vertebra connected to sacral vertebrae is referred to as the cloacal vertebra, which lacks pedestals for the haemal arch, synapophyses, and lymphapophyses [24]. Speculation about the possible relationship between the position of the first haemal arch as a skeletal indicator of sexual dimorphism was previously raised for crocodiles and dinosaurs but ultimately found no confirmation [25,26]. For Komodo dragons, such research is worth considering, In this research, it was concluded that the presence of the haemal arch in Komodo dragons is not gender related. However, age or specific subpopulations were not considered in the analysis. Rakhmiyati and Luthfi described the haemal arch in the Asiatic water monitor (*V. salvator*) as a “processus ventral” and classified the tail vertebrae as procelous [21]. This underscores the need for comprehensive documentation of the presence of these anatomical structures and the correlation of various data.

The blood vessels in the tail are separated from the spinal cord by the tail vertebrae, which is a solution that reduces the risk of injury during fights or tail damage. Blood can be drawn from the animal’s tail at the level of the cloaca, and the tail vein seems to be easily accessible due to the absence of bony space under the vessel, as it is further down the tail. Additionally, the skin and scales are slightly softer in this area. With proper hygiene, this area can be used to administer medications, monitor using Doppler ultrasound for the diagnosis of cardiovascular diseases, assess blood flow in vessels close to the reproductive organs, or evaluate the animal’s pulse. In the methodology of blood sampling from the Komodo dragon, the access to the tail vein is often through lateral puncture of the tail at a depth of 10 to 30 cm [26].

The tail vertebrae in the caudal section display important variation in terms of the number of vertebrae, transverse process structure, shape, and size among different lizard species. However, it is known that the Komodo dragon undergoes a change in its lifestyle from arboreal to terrestrial. Therefore, considering the existing data, it is worthwhile to perform a more detailed morphometry of muscle cross-sections along the entire length of the tail and assess the range of motion for tail flexion associated with specific tail vertebrae, correlating this data with the age of the animal (e.g., utilising geometric morphometrics and structural mechanics). Such studies can be valuable not only for extant species but also in archaeozoological research [27], allowing for a better understanding of survival strategies and population structures in animals. For hatchling Komodo dragons, the tail length-to-snout–vent length ratio (TL:SVL) ranges from 1.7:1 to 2.65:1 [28].

### 4.3. Specific Muscle Anatomy and Function of the Tail

The well-developed muscles represent a structured fibre architecture. This is substantiated by morphometric data, which indicate that the thickest and longest muscle fibres of the *M. Ilio-ischio-caudalis* and *M. caudofemoralis longus* muscles are located in the middle part of the tail. This is also crucial for controlling the tail itself, which is relatively long compared to the body. The balancing character of the tail’s movement, including the shifting of weight from the left to the right limbs, along with the neck’s weight transfer, is evident even when the animal moves slowly.

Studies on the coordination of wavelike movements have been well documented using the model organism of tadpoles (*Rana catesbeiana*). Despite not having vertebrae, their tail myotomes have shown various muscle activity patterns depending on specific behaviours and movements. For instance, during regular swimming, the reduced activity of the terminal myomers optimises movement economy [29]. It appears that the Komodo dragon’s tail tip, even though it has a reduced muscle contribution, serves as a steering and trajectory-correcting tool during its attacks. When swung from the proximal and middle segments, the tail can inflict pain and even injury on prey due to its sharp and hard scales.

The Komodo dragon’s mode of locomotion is also economically justified, with the tail playing a biological role in this regard. Observations from video materials indicate that walking or running Komodo dragons keep the proximal part of their tail raised off the ground, while the distal part remains in contact with the ground. This provides them with greater ease in moving their hind limbs and pelvis. This unwieldy aerodynamic extension helps the Komodo dragon manoeuvre quickly while running to change direction. Based on the curvature of the Komodo dragon’s tail, the understanding of the physiological elevation of the tail’s base in many extinct animal species was rearranged, which has implications for museum exhibits, e.g., *D. carnegii*, Camarasaurus, and Brontosaurus (sauropod dinosaurs)—essentially, all of the Sauropoda elevate the proximal part of the tail, rather than transverse, which is thought to significantly ease egg extrusion [30].

The primary striking force from the tail comes not only from the tail’s base but also from the pelvic part supported by strong hind limbs. When attacking, the animal often positions itself sideways to the prey, allowing it to precisely observe the location and force of its strikes. The Komodo dragon can also overturn prey by undercutting its legs. This restricts the energy expended on prolonged hunting and pursuit of prey and is characterised by a sophisticated feeding technique.

Tail elevation can be a sign of threat or readiness to attack in the Komodo dragon, serving more for defensive rather than offensive purposes. It has been described that these animals nervously move their tails when approaching individuals of the same species while competing for food. When approaching a feeding individual, they can attack with their tails, displaying offensive reactions. This behaviour has also been observed when attempting to remove a Komodo dragon from a cage [31]. The terminal part of the tail essentially shows no significant mobility but serves as a valuable tool for attack. It has been described that adult individuals assume characteristic defensive postures, using their tails as a whip to strike and wound competitors. They can also stand on their hind legs in a straight posture, using the tail for support. This position is adopted when lizards fight by pushing their bodies against each other, a characteristic behaviour for large-sized individuals [32]. Because the tail plays a crucial role in maintaining balance and defence, it is essential to handle the animal by holding its tail at the base, which can be seen in historical photographs [8]. In modern times, immobilising the animal through tail restraint is preferred, avoiding pharmacological sedation [33]. Such restraint must be more cautious in juvenile individuals, whose tails serve more complex locomotor functions. Young individuals use their tails, for instance, to descend from trees by wrapping them around branches (indicating a gripping function), and damage at this stage can lead to severe anatomical imbalances, potentially resulting in elimination in a natural environment.

The entire animal exhibits a wavelike movement during walking, and the tail’s end leaves a sinusoidal trace with regular amplitude [31]. It touches the ground, whereas during running, the tail’s end is raised. These observations suggest that groups of muscles located in the transverse part of the tail are crucial for balance during locomotion, while the striking force and overall posture are supported by the pelvis and proximal tail muscles, providing greater control and strength for tail movement.

The tail also plays a critical protective role for the spinal cord, blood vessels, and nerves. Despite being more susceptible to damage, the terminal part of the tail, due to its anatomical structure with a predominance of connective tissues, has a lower probability of severe infection following injury. The vertebrae in this part of the tail are closely connected, showing little mobility, and the animal itself does not move the tail specifically. The terminal tail vertebrae can be of smaller size, and during the preparation of skeletons, chevron bones are often lost, which results in inaccurate bone structure reconstruction in repositories.

In transversal view, the Komodo dragon’s tail more closely resembles the tails of reptiles living in aquatic or semi-aquatic environments than those of typically terrestrial reptiles [34]. The shape of the tail in adult individuals is laterally flattened, and they exhibit similarities to semi-aquatic species, which does not correspond to the Komodo dragon’s typical terrestrial ecological niche. The sharp dorsal ridge further supports this observation [31]. Extensive studies have been conducted on the tail of the Indian monitor lizard (*V. monitor*), described as a primitive, prehensile, and undulating type of tail, which is similar to the tails observed in fish and amphibians. In deeper sections, the tail of this species loses myomeria. The epithelium is closely connected to the muscles [35]. The sharp dorsal ridge is referred to as a dorsal keel, and it has been identified in many subgenus monitor lizard species, such as empagusia, euprepiosaurus (except *V. juxtindicus*), polydaedalus, soterosaurus, and other species like *V. bengalensis*, *V. dumerilii*, *V. flavescens*, *V. nebulosus*, *V. rudicollis*, *V. caerulivirens*, *V. cerambonensis*, *V. doreanus*, *V. douarrha*, *V. finschi*, *V. indicus*, *V. jobiensis*, *V. lirungensis*, *V. melinus*, *V. obor*, *V. rainerguentheri*, *V. semotus*, *V. yuwonoi*, *V. zugorum*, *V. boehmei*, *V. acanthurus* [28], and others, mostly aquatic or semi-aquatic species [36]. The Komodo dragon also possesses a dorsal keel, although its morphology changes along the tail’s length and is less pronounced in the proximal part of the tail. Swimming reptiles are characterised by long tails that serve as the main propulsive force, while lizards with short tails rely more on their limbs for locomotion [4].

The anatomy of the tail vertebrae in fossil *V. komodoensis* species seems to mirror that of contemporary individuals. However, this species exhibits different geographic distributions across isolated islands in eastern Indonesia and between Java and Australia, as well as the Timor islands. These anatomical similarities support the hypothesis that gigantism is not a result of island evolution. It is most likely that this species expanded westward from mainland Australia and subsequently experienced phyletic gigantism [37]. In phylogenetic analyses of monitor lizards, researchers have employed morphological data, including aspects related to size, such as tail vertebrae. To better understand the evolution and function of the tail, authors emphasise the need for an interdisciplinary approach. Particularly, they point out that tails usually serve multiple functions [4,38].

### 4.4. Multifunctionality of Fat Storage in the Komodo Dragon’s Tail

Adipose tissue deposition in the tail of the Komodo dragon initiates behind the cloacal region, initially forming thin layers between different muscle sections within the septa separating the muscle masses. The primary fat deposits are situated between the *M. caudofemoralis longus* and *M. ilio-ischio-caudalis* muscles, creating crescent-shaped layers of adipose tissue along the sides of the tail, beneath the transverse processes of the vertebrae. Further along the tail, adipose tissue begins to accumulate closer to the spinous processes and above the transverse processes.

The integrated perspective addresses crucial issues related to the tail’s utility and biology, including its potential for regeneration, functional morphology, and sensorimotor control. At times, it is even referred to as the “fifth limb,” highlighting its importance in terms of thermoregulation and energy storage, a concept that has been adopted by bio-robotics [38]. Many species demonstrate an allocation of resources and trade-offs between locomotor functions, fat storage, and regeneration post-autotomy. Thus, for some species, the ability to regenerate the tail might be considered an unfavourable biological investment. The tail has a high surface-to-volume ratio and can act as a thermal window to release excess heat. The Komodo dragon, while lacking the ability for tail autotomy (self-amputation), serves a crucial function in storing fat tissue, which has consequences for the species’ biology. In this species, fat tissue is significantly reduced in other body areas, as small abdominal fat bodies are observed during dissection. Therefore, the fat stored in the tail represents the majority of adipose stores in the body [39].

Heat transfer to the body surface is more efficient in lizards with an increased surface area, especially those with long tails. Heliothermia and thigmothermia are of utmost importance for lizards with long tails, where the animal gains heat through direct contact with the substrate and solar radiation. For giant species, endogenous heat is lost proportionally to its production, and this heat dissipation through body coverings prevents overheating. Moreover, larger species require more time to accumulate heat. Komodo dragons maintain their preferred body temperature at around 35 °C, and their thermoregulatory habits depend on their size and microenvironment. They transition into a more sedentary lifestyle as they age. Thermal imaging studies have shown no significant temperature difference between the head, body, and tail, with the tail temperature remaining slightly higher during nighttime cooling and early mornings [40].

It has been indicated that Komodo dragons actively regulate their body temperature throughout the day, with larger individuals taking longer to heat up and cool down [41] and have an inability to reach a thermal equilibrium overnight, as they lose heat due to thermal inertia and low thermal conductivity [42]. This experiment should involve measurements taken on the lower part of the tail, considering the presence of larger blood vessels in the nearby area and the assessment of thermal conductivity. These results already demonstrate that with age and a change in lifestyle, the accumulated fat is more useful for these species because they become more dependent on daily temperature fluctuations and fat metabolism. Komodo dragons are ectothermic predators [43]. In their natural tropical ecosystem, they exhibit strong seasonal patterns of female reproductive activity and hatching of offspring. The dry season favours their locomotor activity and daily movement pace. No correlation has been observed between environmental differences and somatic growth rate and body condition [44].

Storing fat in the tail not only helps in explaining the thermoregulatory metabolism [45] but also reveals the energetic balance in hunting strategies. Sedentary and ambush predators store more fat, allowing them to remain reproductive year-round, independent of food availability. These are the three key issues that need to be addressed concerning the Komodo dragon: metabolic homeostasis dependent on stored fat is crucial for thermoregulation and animal activity, including proper behavioural displays and reproduction. Low-density lipoproteins (VLDLs) play a significant role in reproduction and are used during vitellogenesis (egg yolk formation) [46].

The presence of fat in the Komodo dragon’s tail has implications beyond biology. It is an important conservation issue. Population dynamics have been observed to shift towards evidence of energetic constraints. Some *Varanus* populations show a shift in the frequency towards smaller and lighter individuals. A subpopulation on Gili Motang exhibited nearly half the somatic growth rate compared to other groups, reflecting a population vulnerable to food scarcity, which may necessitate additional protection and support from humans [47]. This could also lead to significant reproductive problems affecting the species’ life history [48].

Additionally, historical data have confirmed the impact of human activities on monitor lizard populations due to the extraction of fat from their tails. In 1927, reports indicated the use of tail fat from *V. Salvator* by the Chinese for burn treatments. This tradition was later replicated in Indonesia and Singapore to produce “minak minjawak” (Indonesian: lizard oil) [31]. A similar practice of obtaining a therapeutic substance from the tail of *V. bengalensis* was described among the ‘Adi’ tribe. They would leave the whole tail to dry, then macerate and grind it into a powder. This powder was used as a veterinary feed additive for cattle to improve their condition and health and for wound treatment. Tail fat from monitor lizards was also used according to beliefs in human medicine, such as massage balm, ointment for hemorrhoids, rheumatism, pain relief, and for burns (not only topically) [49].

Awareness of the presence of fat in the tail of the Komodo dragon can also be useful for collectors at the Natural History Museum. Previously unremoved tail fat may remain on bones during preparation, attracting insects that can damage such museum collections. The Komodo dragon stores essential mineral compounds along with fat necessary for the proper functioning of all organs. It has been demonstrated that there is a high accumulation of calcium, phosphorus, potassium, sodium, magnesium, iron, and zinc in the adipose tissue relative to their levels in the blood (Table 1 and Table S1). Furthermore, they accumulate elements that provide insight into the local natural environment and can serve as bioindicators. The profile of fatty acids can help identify dietary stress. For reptiles, the onset of starvation is marked by an increase in the unsaturated fatty acid index and an increase in the proportion of linoleic acid to palmitic acid, oleic acid to palmitic acid, and arachidonic acid to the total fatty acid concentration [50,51]. In Komodo dragons, the composition of fatty acids is as follows: saturated fatty acids account for 44.34%, monounsaturated fatty acids for 45.09%, and polyunsaturated fatty acids for 11.33%. The predominant fatty acids in the sample were oleic acid (34.94%) and palmitic acid (26.61%) (Table 1 and Table 3).

### 4.5. Observations on Cloacal Glands and Spinal Cord

The fatty–waxy substance is produced in the cloacal glands, which histologically, contain both serous and mucous units. Considering the topography and anatomical structure, particularly the wide outlets, symmetrical placement, and well-developed musculature, it can be assumed that secretion occurs during the compression of fecal masses on the surrounding structures during egg laying. It is not excluded that they are related to chemical communication, for example, associated with fecal excretion and territorial marking.

Reptiles exhibit significant anatomical diversity in their central nervous system, which is reflected in the differences in tail structure and the way the animal moves. Lizards, for example, are among the animals that move with a predominance of trunk and limb muscles, using diagonal limb patterns. In reptiles that have lost their limbs, the spinal cord does not represent cervical and lumbar enlargements. In reptiles, the boundary between gray and white matter is less distinct than in mammals and birds. White matter forms the dorsal, lateral, and ventral columns. Gray matter in reptiles has distinct dorsal and ventral horns [54]. It has been observed that in Komodo dragons, the dorsal horns in the spinal cord of tail vertebrae are smaller. White matter predominates over gray matter, forming a heart-shaped cross-section of the cord. This may have consequences in terms of a lower number of sensory processing cells in this region. The Komodo dragon possesses the ability for rapid movement and powerful tail strikes, which may be facilitated by its relatively large motor neurons and a substantial amount of myelinated nerve fibres in the thicker layer of white matter. This structure promotes faster signal transmission and impulse conduction to the muscles. Furthermore, the good blood supply in the spinal cord supports physical performance and metabolic efficiency. Additional research on the spinal cord could help in understanding the specific adaptations of this species.

## 5. Conclusions

The Komodo dragon possesses a remarkably long tail, extending to approximately half the length of its body, supported by a linear skeleton comprising 60–86 vertebrae. This anatomical feature fulfills crucial ecological roles, especially aiding juveniles in their arboreal lifestyle and serving as a counterbalance for the massive front body weight in adults, facilitating balance in movement given their gigantic size. The tail skeleton’s configuration includes haemal arches beginning after the first tail vertebrae, surrounding blood vessels. The absence of haemal arch in the first caudal vertebrae may require further investigation with a larger sample size since it is valuable information about phylogenetic relationships and could be a determining factor for a subpopulation or its relatives. The tail’s shape provides this animal with better manoeuvrability in water, even though it is primarily a terrestrial species, thanks to the presence of a dorsal keel, most prominent in the transverse part of the tail. During movement, the Komodo dragon uses the tail for steering and correction, allowing it to easily change direction. Muscularly, the proximal part of the tail exhibits robust development, prominently featuring the *M. caudofemoralis longus*, along with the *M. Ilio-ischio-caudalis*.

Based on observations and morphological characteristics, it is evident that the tail is multifunctional and its significance extends beyond locomotion. It is particularly adapted for fighting and defence. In confrontations with other individuals, the Komodo dragon assumes a characteristic defensive posture in which the tail serves as support when its body is upright. The force applied to the target is generated by the extensive tail muscles, along with the involvement of the pelvic girdle. These powerful strikes are dampened by muscle layers with varying fibre architecture, along with massive vertebrae, minimising the risk of spinal cord and vascular trauma. Thus, the tail is pivotal not only for locomotion but also for maintaining overall body posture.

Adipose tissue storage in the tail represents a critical biological investment for the Komodo dragon. Unlike some lizard species, the Komodo dragon lacks regenerative abilities in its tail, underscoring the importance of stored fat reserves. These adipocytes, averaging 54.37 µm in diameter, form solid layers separated by connective tissue. Fat reserves are primarily located in the tail, as other body regions have minimal fat deposits. The context of adipose tissue storage can be justified as a biological investment for the organism. The greatest benefits are transferred to reproductive and survival capacities. The latter is associated with thermoregulation. The large tail surface area acts as an effective thermal window to dissipate excess heat. The accumulated fat enables the animal to maintain a relatively constant body temperature despite daily temperature fluctuations and seasonal climate changes. As individuals age, the storage of reserve fat in the tail takes on a more important role, influencing their reproductive activity based on the environment. The animal can utilise stored fat in a planned metabolic strategy, which, to some extent, makes its reproductive season independent of the food-limited time of the year. The tail’s fat reserves are crucial for thermoregulation and energy storage, supporting reproductive and survival strategies.

To maintain efficient thermoregulation, saturated fatty acids play a crucial role. They enable the animal to elevate its temperature. Due to the substantial impact of tail fat on the progression of all biochemical processes in the body and its known influence on the quality and composition of the diet, dietary management is especially important in treating lizards. Many functions of tail fat have yet to be fully explored, although it is known to serve an immunological and endocrine function in other animals. This biological relevance of tail fat provides Komodo dragons with a competitive advantage in their environment and may have been a reason for their increased adaptability.

In addition to being an energy source, the accumulated tail fat is rich in essential mineral elements such as calcium, phosphorus, potassium, sodium, magnesium, iron, and zinc. The analysis of the biochemical composition of this fat can provide insights into environmental contamination with heavy metals and other factors. Furthermore, the proportion of fatty acids in the tail fat indicates the animal’s nutritional status and condition, pointing to possible dietary stress. All of this data are important both for the Komodo dragons in their natural habitat and for those kept in zoological gardens.

An important implication is also the use of tail fat in ethnomedicine, which raises concerns about the potential exploitation of these tissues and the Komodo dragon itself as targets of crimes against nature. However, it is important to note that the fat from this animal’s tail is unlikely to possess exceptional medicinal properties, and consuming it could potentially be harmful due to the risk of accumulating heavy metals.

Looking forward, future research on the Komodo dragon’s tail should expand into several key areas. Comprehensive morphometric studies of tail vertebrae and muscles, integrated with age correlations, would deepen our understanding of structural adaptations. Investigations into thermoregulation dynamics, particularly heat dissipation from the tail’s ventral side, remain pivotal. Detailed analyses of fatty acid profiles and histological examinations of spinal cord tissues could unveil additional metabolic insights. Biomechanical inquiries into defensive and offensive behaviors would shed light on the tail’s functional mechanics under various stress conditions. Exploring hormonal regulation mechanisms governing adipose tissue storage in the tail could uncover adaptive strategies linked to environmental variability. Comparative studies with other large reptiles would further elucidate evolutionary adaptations specific to the Komodo dragon’s tail, offering broader insights into saurian physiology and behavior.

## Figures and Tables

**Table 1:** Content of selected minerals in the tail fat tissue sample.

Minerals	Content [µg/g Fat]
Ca	1732 ± 187
P	1359 ± 59
K	201.6 ± 12
Na	53.37 ± 2.13
Mg	27.08 ± 3.02
Fe	9.52 ± 0.42
Zn	3.74 ± 0.27
Cu	0.105 ± 0.438
Cd	0.034 ± 0.0067
Hg	0.0012 ± 0.00004
Pb	<0.005

**Table 2:** The components of fatty acids from tails fat tissue determined using gas chromatography.

Area [g/100 g] %	C_n_:n	Fatty Acid
0.05902	C6:0	Caproic acid
0.09023	C18:3 n6	Gamma-linolenic acid (GLA)
0.10943	C8:0	Caprylic acid
0.14130	C20:5	Eicosapentaenoic acid (EPA)
0.16512	C17:1	Heptadecenoic acid
0.17593	C18:3 n3	Alpha-linolenic acid (ALA)
0.19400	C20:3 n3	Eicosatrienoic acid (n-3)
0.19803	C20:2	Eicosadienoic acid
0.21951	C20:0	Arachidic acid
0.26156	C22:1	Erucic acid
0.29719	C15:0	Pentadecanoic acid
0.46382	C:17:0	Margaric acid
0.76226	C20:1	Eicosenoic acid
1.40764	C14:0	Myristic acid
4.42079	C16:1	Palmitoleic acid
4.53312	C18:1trans	Oleic acid (trans)
6.40996	C4:0	Butyric acid
8.76253	C18:0	Stearic acid
9.77179	C18:2	Linoleic acid
26.61068	C16:0	Palmitic acid
34.94609	C18:1cis	Oleic acid (cis)

**Table 3:** Lipid properties in animal adipose tissue [52,53].

Composition of Adipose Tissue in Animals	Komodo Dragon (*V. komodoensis*), Tail Fat	Nile Crocodile (*Crocodylus niloticus*), Tail Fat	Bactrian Camel (*Camelus bacterianus*), Camel Hump	Bactrian Camel (*Camelus bacterianus*), Tail Fat
Predominant fatty acids	C16:0 (26.61%) C18:1cis (34.94%)	C16:0 (28.7%) C18:1c9 (30.8%)	C16:0 (18.76%) C18:0 (23.23%) C18:1 n-9 cis (34.91%)	C16:0 (17.98%) C18:0 (17.18%) C18:1 n-9 cis (39.96%)

## Data Availability

No other details regarding where data supporting the reported results can be found, including links to publicly archived datasets analysed or generated during the study.

## Supplementary Materials

The following supporting information can be downloaded at: https://www.mdpi.com/article/10.3390/ani14152142/s1, Table S1. Data on blood parameters were obtained from the Ragunan Zoological Garden publication (Hak Cipta IPB, 2012, Bogor) [55], as well as from data collection archieved by the Department of Animal Anatomy, Histology, and Pathomorphology, named after Academician Vladimir G. Kas’janenko at the National University of Life and Environmental Sciences of Ukraine. Additional data were obtained from the literature on other species, as well as data based on the literature review, and non-representative data with no clinical value were also included [56,57,58,59,60,61,62]. N/a—not indicated.
