# Supplementary material for: Biological Significance of the Komodo Dragon’s Tail (Varanus komodoensis, Varanidae)
Source: Animals (Basel). 2024 Jul 23;14(15):2142. doi: 10.3390/ani14152142 (PMC11311070; doi:10.3390/ani14152142)
Supplement: Supplementary file 1 [file animals-14-02142-s001.zip › animals-3029664-supplementary.pdf]

**Blood values in different species**	*V. komodoensis*	*V. niloticus*	*V. prasinus*	*V. exanthematicus*	*Crocodylus intermedius*	*Iguana i. iguana*
WBC (Leukocytes)	(6,53±9,47) $\times 10^3$ $/mm^3$ 0.7-12.5 x 10^9/L	1.96)x10^9/L	9.717±5.934 cells/μL	(9.57±1.68) 10^9/L	304-3690 cells/μL	n/a
NEU (Heterophils)	(3,478±4,972) $\times 10^3$ $/mm^3$ 0.08-0.58 (fraction from leukocyte)	21.72%	3.717±3.313 cells/ μL	9.50±0.85)10^9/L	300-9595 cells/ μL	0.35-5.2 x 10^9/L
LYMPH (Lymphocytes)	(2,959±4,694) × 10^3 /mm^3 0.35-0.82 (fraction)	67.33%	3.733±3.313 cells/ μ L	n/a	304-3690 cells/mikrol	0.5-5.5 x10^9/L
Mono (M onocytes)	(0,096±0,187) $\times 10^3$ $/mm^3$ 0.0-1.5 x 10^9/L	9.28%	401±737 cells/ μ L	1.50±0.22)10^9/L	0-346 cells/mikrol	0.0-0.1 x 10^9/L
EO (Eozyno philis)	(0,00) $/mm^3$ 0.0 x 10^9/L	1.00%	n/a	1.67±0.33)10^9/L	0-622 cells/mikrol	0.0-0.3 x10^9/L
Baso (Basophilis)	(0,00) $/mm^3$ 0.00-0.6 x 10^9/L	0.67%	n/a	0.00±0.00)10^9/L	n/a	0.0-0.5x10^9/L
TP (Total protein)	(10,19±3,39) g/dL 7.2-10.6 g/dL	72,6 g/L (74.5 g/L)	94,9 g/L (74±24 g/L)	7.07±0.39)g/L	3.9-10.2 g/dL	50-78 g/L
ALB (Albumin)	(2,51±0,39) g/dL 2.0-4.5 g/dL	28.0 g/L	2.9±0.8 g/dL	n/a	1.4-3 g/dL	21-28 g/L
GLO (Globulins)	(7,68±3,07) g/dL 2.2-8.4 g/dL	46.5 g/L	5±1.4 g/dL	n/a	92-186 mg/dL	25-43 g/L
AST (Asparagine aminotransferase)	(49,39±20,71) IU/L 10.0-39.0 IU/L	6 IU/L	10 IU/L (26±17 IU/L)	n/a	25-260 IU/L	5-52 IU/L
ALT (Alanine aminotransferase)	(45,39±27,88) IU/L	4 IU/L	14 IU/L (10±3 IU/L)	n/a	11.5-100 u/L	5-68 IU/L
Urea	(13,53±5,88) mg/dL 0.4-1.8 mmol/L	0.5 mmol/L	0,6 mmol/L(1.07 ±0.71 mmol/L)	n/a	n/a	n/a
UA (Uric acid)	170-1620 μmol/L	n/a	422.3±392.6 mmol/L	n/a	1.8-7.9 mg/dL	70-140 mmol/L
CREA (Creatinine)	(0,29±0,11) mg/dL 20-90 μmol/L	41,1 μmol/L	26.52±17.68 mmol/L	n/a	0.4-0.7 mg/dL	n/a
TG (Triglycerides)	n/a	0,53 mmol/L	0,09 mmol/L	n/a	n/a	0.6-7.8 mmol/L
TC (Total cholesterol)	n/a	2,64 mmol/L	1,53 mmol/lL(8.6 8±4.31 mmol/L)	n/a	164-357 mg/dL	n/a
LDL-C (LDL cholesterol)	n/a	0,05 mmol/L	n/a	n/a	n/a	n/a
HDL-C (HDL cholesterol)	n/a	n/a	0,01 mmol/L	n/a	4-47.1 u/L	n/a
TBIL (Total bilirubin)	n/a	0,3 μmol/L	0,8 μmol/L	n/a	n/a	n/a
K (Potassium)	3.2-564 mmol/L	5,2 mmol/L	3,9 mmol/L	n/a	n/a	n/a

		(3.8 mmol/L)	(4,1±0.8 mmol/L)			
Na (Sodium)	158-174 mmol/L	154 mmol/L (118.9 mmol/L)	148 mmol/lL(154 ±11 mg/dL)	n/a	n/a	n/a
Ca (Calcium)	3.10-4.22 mmol/L	n/a	n/a	n/a	n/a	n/a
P (Phosphorus)	1.15-2.60 mmol/L	n/a	n/a	n/a	n/a	n/a
Cu (Copper)	10.2-20.8 µmol/L	n/a	n/a	n/a	n/a	n/a
Mg (Magnesium)	1.02-1.68 µmol/L	n/a	n/a	n/a	n/a	n/a
Fe (Iron)	11-22 µmol/L	n/a	n/a	n/a	n/a	n/a
Zn (Zinc)	31.8-163.7 µmol/L	n/a	n/a	n/a	n/a	n/a
LDH (Lactate dehydrogenase)	108-2139 IU/L	101 IU/l	68 IU/l	n/a	16-178 mg/dL	n/a
AMY (Amylase)	n/a	1530 IU/l	7580 IU/l	n/a	n/a	n/a
GLU (Glucose)	6.7-14.4 mmol/L	6.2 mmol/L (8.6 mmol/L)	4,4 mmol/lL (6.98±2.75 mmol/L)	n/a	n/a	n/a

Table S1. Data on blood parameters were obtained from the Ragunan Zoological Garden publication (Hak Cipta IPB, 2012, Bogor) [50], as well as from data collection archieved by the Department of Animal Anatomy, Histology, and Pathomorphology, named after Academician Vladimir G. Kas'janenko at the National University of Life and Environmental Sciences of Ukraine. Additional data were obtained from the literature on other species. As well as datas based on literature review, non-representative ones with no clinical value were also included [51-57]. N/a - not indicated.
